# Phase and Index of Refraction Imaging by Hyperspectral Reflectance Confocal Microscopy

**DOI:** 10.3390/molecules21121727

**Published:** 2016-12-16

**Authors:** Stefano Selci

**Affiliations:** Consiglio Nazionale delle Ricerche-Istituto dei Sistemi Complessi—CNR-ISC, Via Fosso del Cavaliere 100, 00133 Rome, Italy; Stefano.Selci@isc.cnr.it; Tel.: +39-06-4993-4167

**Keywords:** hyperspectral microscopy, scanning confocal microscopy, index of refraction, red blood cells

## Abstract

A hyperspectral reflectance confocal microscope (HSCM) was realized by CNR-ISC (Consiglio Nazionale delle Ricerche-Istituto dei Sistemi Complessi) a few years ago. The instrument and data have been already presented and discussed. The main activity of this HSCM has been within biology, and reflectance data have shown good matching between spectral signatures and the nature or evolution on many types of cells. Such a relationship has been demonstrated mainly with statistical tools like Principal Component Analysis (PCA), or similar concepts, which represent a very common approach for hyperspectral imaging. However, the point is that reflectance data contains much more useful information and, moreover, there is an obvious interest to go from reflectance, bound to the single experiment, to reflectivity, or other physical quantities, related to the sample alone. To accomplish this aim, we can follow well-established analyses and methods used in reflectance spectroscopy. Therefore, we show methods of calculations for index of refraction *n*, extinction coefficient k and local thicknesses of frequency starting from phase images by fast Kramers-Kronig (KK) algorithms and the Abeles matrix formalism. Details, limitations and problems of the presented calculations as well as alternative procedures are given for an example of HSCM images of red blood cells (RBC).

## 1. Introduction

The hyperspectral reflectance confocal microscope (HSCM) was realized a few years ago at CNR-ISC (Consiglio Nazionale delle Ricerche-Istituto dei Sistemi Complessi). The instrument set-up and experimental details have been already presented [1] and discussed [2]. The main application of this HSCM, working in a broad VIS and NIR spectral range, has been within biology, investigating for instance many types of cells, like the acquisition of multi-dimensional spectral images of human melanoma cells [2], or the discrimination of HaCaT and melanoma cells in a co-culture model, for which reflectance data have shown good matching between spectral signature and cells’ nature or evolution [3]. Such a relationship has been demonstrated [4] mainly with statistical tools like Principal Component Analysis (PCA), a very common approach for hyperspectral imaging [5,6,7], together with similar statistical tools. However, the point is that reflectance data contains much more information that can be gathered. There is in fact an obvious interest to overcome the limitations of reflectance, tied to the specific experiment, and approach reflectivity, related to the sample alone. To accomplish the desired results, we can follow old and well-established routes used in reflectance spectroscopy, like the one described in a classical solid-state physics reference book [8]: because of causality, it is possible to add a related piece of information transforming the experimental local reflectance into equally local wavelength dependent phase spectra, which is the so-called Kramers-Kronig (KK) analysis. Eventually, other equations exist to use both parts to achieve the entire complex index of refraction of the observed specimen. This latter part makes a very simple use of the Abeles matrix method [9], similar to methods that have been extensively used in very different contexts as well [10].

The final aim is the derivation of local thickness as well the index of refraction of a selected object, in this case an erythrocyte, trying to translate results of the observation into inherent quality and quantity of the observed sample not dependent on the specific set-up or observation event.

While KK analysis of spectroscopic images and derivation of index of refraction parameters are not at all new in biology, nor within microscopy-based techniques, the contribution presented here is believed to be an original one because with very few exceptions hyperspectral reflectance confocal microscopy is not widely used [11]. Other ways to obtain similar results are either missing local information (and thus they are not able to produce maps) or require rather more cumbersome experimental procedures and theoretical considerations, like in the case of interference microscopy set-ups that require far more complex equipment and multiple comparisons for the final results. These are many examples of techniques currently available for which spectroscopic elaborations are possible.

For instance, a phase and index of refraction (RI) model is studied in fibroblast and mitochondria cells [12], with a Linnik microscope interferometric set-up, using a fixed wavelength laser, similar to the OCT arrangement [13] with similar purposes. The importance of RI measurements for biology is well underlined in a work that it is one of the first introducing Quantitative Phase Microscopy (QPM) [14], presenting results for the thickness and RI of airway smooth muscle (ASM) cells. Due to the difficulties of QPM to decouple evaluation of thickness and RI, both involved in the evaluated optical path difference (OPD), a further procedure is presented based on digital holographic microscopy (DHM) [15] founded on a dual wavelength observation, as for other successive works [16,17]. Here, the KK equation is part of the needed formalism. Another work [18] uses an approach very similar to the one presented here: different wavelengths are available by using a white source (a xenon lamp), by a suitable band-pass optical filter, and each component OPD is measured and used for evaluation of RI. However, in this case a wide-field microscope is used, instead of a HSCM confocal arrangement, and only one wavelength can be selected at one time, so no hyperspectral images are available: a full hyperspectral recording could be useful for future applications like serial diagnoses and unattended identifications of unknown samples, without the need of a-priori knowledge of the right spectral region. Another result using QPM technique [19] underlines very well the importance to have a method to quantify the RI on the very local scale. In this case, the observed increase in nuclear refractive index is related to cells becoming malignant. The DNA proliferation (that has UV specific optical absorption) can be related to that change.

The above is a very important point: the observation in one spectral window can be always related to the properties in other spectral regions. Obviously, we are speaking of general principles of causality, which imply dispersion rules and with the KK formalism that describes what happens behind the scene. This point will not be discussed further here, but it is an interesting topic to be developed in subsequent works.

All previous techniques have been expressed eventually in the very broad concept of Quantitative Phase Imaging (QPI) after the seminal work of Popescu [20], as can be further appreciated in a later review [21] and many practical applications [22,23]. Quite interestingly, QPI is a mature multi-technique viewpoint which approaches, however, formally do not include techniques based on spectral reflectance, like the one presented here, despite the very old legacy in that respect.

For many years phase retrieval and KK analysis have had broad application outside of microscopy. In a recent work [24] phase and RI are evaluated for DNA nucleotides under variable magnetic fields. Another work [25] has a very similar approach to the one presented here, except that there is no local spectroscopy: reflectance is obtained from an entire culture cell. The invoked fundamental procedures are very similar. What it is missing there is the opening to the most complete generalization of the sample structure: a problem solved here by introducing a multilayer approach as a way to generalize by modeling via sample stratifications. Another topic, instead, is not considered here with respect to the last cited work, i.e., the tails problem (regions where no direct data are available) in the evaluation of the KK contributions, a well-known issue [26] that has been omitted here for brevity at this stage of the discussion, but which has to be included in a future enhancement of this work. A refined example of usage of KK analysis and tails’ inclusion is in a study for a very wide spectral range, from THz to far UV, applied to human skin [27], that confirms the increasing importance of such mathematical methods in biology. A very recent work [28] on colorectal dysplasia studied RI with fixed angle and multiple wavelengths reflectometry (no microscopy was involved) finding that a significant index contrast exists in samples between normal and pathological states, reinforcing the potential use of RI as a marker.

Finally, it is worth stressing that RI contrast is the major source of confocal microscopy imaging, also for biological tissues of moderate thickness, as compared to scattering and absorption [29]. This underpins the need to fully explore the Fresnel formalism, consequently including the consideration of wavelength-dependent optical properties expressed like mathematical complex entities, for both the phase and the absorption description. Despite the fact that very often the observation of biological objects under microscopic observation is formulated in terms of scattering, in this paper scattering is completely neglected considering that, in many situations, a great part of the confocal signal is backscattered in the same direction of the incident wave. This is an approximation, of course, but this is a viewpoint that merits to be fulfilled until it produces consistent results. Scattering can be taken into consideration later in order to explain all the signal parts that cannot be expressed in terms of Fresnel equations, but this goes beyond what is presented here.

## 2. Materials and Methods

### 2.1. Mathematical Basis of Reflectance Manipulation

The ISC-CNR HSCM confocal microscope used here is fed by a supercontinuum laser source working within the 0.5 µm and 2.4 µm range, although in practice it is limited to about 1.6 µm. While the reflectance spectra associated to each image point have enough information to perform sensitive analyses of cells and tissues simply by direct comparison, the required specimen parameters (e.g., optical constants or cells thickness) are hidden within broad spectral features. Because reflectance spectra have available wavelengths in a continuous way by using this HSCM, KK based elaborations are possible.

A reflectance spectrum is associated with every image point and, therefore, the number of needed calculations is both relevant and demanding for an ordinary desktop computer. Moreover, the results are likely to be displayed as fast as possible to be quickly analyzed within the experimental session. Therefore, an original algorithm for the KK part is also presented allowing faster computations of the results.

The sample used as an example and reported here is a very simple image of red blood cells (RBC) withdrawn from the author’s finger and roughly deposited on a microscope glass slide. The example of data mangling is shown after explanation of the various algorithms and mathematical transformations started from reflectance data, including the associated phase image by KK calculus. A quite general, although here simplified, layered dielectric function modeling is finally associated, allowing the derivation of an effective complex index of refraction ${\tilde{n}}_{eff}$. While following the given model, numerical fitting of data is possible by a multi-parametric best-fit of the spectra, and the number crunching approach by itself hides the physical insight: as demonstrated below, fine results can be obtained with a very small effort, simply reasoning on the models’ predictions. Eventually, a practical example will be given with a further simplified approach. Because of its practical usefulness, a possibly original algorithm that speeds-up considerably the KK transformation of reflectance data, but usable in many other contexts as well it will be also explained. Completing the modeling with numerical data fit, which is unavoidable for the most complete determination of all the involved parameters, will be the aim of a subsequent paper.

The HSCM is able to collect a reflectance spectrum for every point of the explored sample. If a signal is returned it means that there is a RI discontinuity at that point. The term RI (widely used biological spectroscopy) will be extensively replaced with the standard notation for the refraction index $\tilde{n}\left(\omega \right)=n\left(\omega \right)+i\;k\left(\omega \right)$, where the explicit dependence on the frequency has been added, often used with the more common wavelengths notation “*λ*”. The simply collected reflectance needs at least to be normalized with some reference spectrum before it can be used for any calculation to get rid of the microscope’s instrumental transfer function. How this is done will be explained presenting real data.

Following standard notations [8,9], we can generally write the reflectivity as the absolute square of the reflectivity amplitude as $R\left(\omega \right)=\tilde{r}\left(\omega \right){\tilde{r}}^{*}(\omega )$.

On the one side, we can write the reflectivity amplitude in terms of modulus and phase:

(1) $$\tilde{r}(\omega )=\rho \left(\omega \right)e^{i\theta \left(\omega \right)}$$

so that $R(\omega )=\rho^{2}(\omega )$.

On the other side, the reflectivity amplitude is related to the (possibly local) refraction index discontinuity. We are almost at near normal incidence so we can use the Fresnel equation [9] for the normal incidence case:

(2) $$\tilde{r}\left(\omega \right)=\frac{\left({\tilde{n}}_{2}\left(\omega \right)-n_{1}\left(\omega \right)\right)}{\left({\tilde{n}}_{2}\left(\omega \right)+n_{1}\left(\omega \right)\right)}$$

where ${\tilde{n}}_{2}=n_{2}+i\;k_{2}$. At variance with respect to Wooten [8] in Equation (2) the incoming radiation is propagating in a medium of generic index of refraction $n_{1}\left(\omega \right)$, instead of vacuum or air, because practical applications of microscopy demand so. Of course, that medium cannot have absorption of any sort to allow free propagation. The two simple Equations (1) and (2) are the connection blocks for all the following discussion.

As it is well known [8], causality requires a very precise relationship between the real and imaginary parts of Equation (1), that is the dispersion rule that binds the two parts. Because $\ln\tilde{r}(\omega )=\ln\rho \left(\omega \right)+i\theta \left(\omega \right)$:

(3) $$\theta \left(\omega_{0}\right)=-\frac{1}{\pi }℘\int_{-\infty }^{\infty }\frac{\ln\rho \left(\omega \right)}{\omega -\omega_{0}}\;d\omega$$

It can be shown [8] that analyticity of the complex path of the electric disturbance $\vec{E}$ suggests a more robust dispersion rule:

(4) $$\theta \left(\omega_{0}\right)=\frac{\omega_{0}}{\pi }\int_{0}^{\infty }\frac{\ln\left[R\left(\omega \right)/R\left(\omega_{0}\right)\right]}{\omega_{0}{}^{2}-\omega^{2}}\;d\omega$$

As already pointed out, the form of Equation (4) implies that arbitrary multiplication factors of the reflectivity R(ω) are removed in the ratio and that any divergence is also removed. The standard procedure [25] at this point is to equate the reflectance complex amplitude (1) with the (normal incidence) Fresnel Equation (2). Taking into account Equation (4), it is possible to solve explicitly for $n_{2}$ and $k_{2}$:

(5) $$\left\{ \begin{array}{l} n_{2}=\frac{n_{1}(1-R(\omega ))}{1+R(\omega )-2\sqrt{R(\omega )}\cos(\theta )}\\ k_{2}=\frac{2n_{1}\sqrt{R(\omega )}\sin(\theta )}{1+R(\omega )-2\sqrt{R(\omega )}\cos(\theta )} \end{array}\right.$$

Equations (5) have been also recently reported [25], but in this case $n_{1}$, the index of refraction of the medium from which radiation is propagating, is explicitly included. In any case, Equations (5) are not general enough to describe correctly some typical microscopic observations of biological samples.

Suppose we have cells deposited on a glass slide. The microscope beam can be propagating in air, or, instead, transmitting through a liquid, like a Dulbecco medium [3,4]. We are thus facing at least a three layer system where the cell, the intermediate layer between two semi-infinite media, can be represented by a homogenous (complex) index of refraction. We could also want to further refine the model decomposing our sample into finer layers, for instance to simulate a more complex structure. Therefore, we need to refine Equation (2) by a more general expression. The more general expression for the reflectance amplitude, within a Fresnel-like scheme, can be found with the aid of the Abeles matrix formalism [9]. The reflectance amplitude, s-polarization, can be written:

(6) $$\tilde{r}=\frac{\left(m_{11}+m_{12}p_{l}\right)p_{1}-\left(m_{21}+m_{22}p_{l}\right)}{\left(m_{11}+m_{12}p_{l}\right)p_{1}+\left(m_{21}+m_{22}p_{l}\right)}$$

where $m_{ij}$ are the elements of a 2 × 2 characteristic matrix of a stack of *l−2* layers, obtained by matrix multiplication of matrices, each one for layers of arbitrary thicknesses and optical properties, bound between two infinite media, and $p_{k}=\sqrt{\varepsilon_{k}/\mu_{k}}\cos\left(\vartheta_{k}\right)$, at an arbitrary angle of incidence. The explicit dependence on the frequency has been omitted for brevity. Now, we are interested in normal incidence, and the index of refraction of the medium for the incoming beam has to be real, so we can rewrite Equation (6) in a simplified form:

(7) $$\tilde{r}=\frac{\left(m_{11}+m_{12}{\tilde{n}}_{3}\right)n_{1}-\left(m_{21}+m_{22}{\tilde{n}}_{3}\right)}{\left(m_{11}+m_{12}{\tilde{n}}_{3}\right)n_{1}+\left(m_{21}+m_{22}{\tilde{n}}_{3}\right)}$$

Rearranging the terms:

(8) $$\tilde{r}=\frac{n_{1}-\frac{\left(m_{21}+m_{22}{\tilde{n}}_{3}\right)}{\left(m_{11}+m_{12}{\tilde{n}}_{3}\right)}}{n_{1}+\frac{\left(m_{21}+m_{22}{\tilde{n}}_{3}\right)}{\left(m_{11}+m_{12}{\tilde{n}}_{3}\right)}}$$

Equation (8) is formally equivalent to Equation (2) if one introduces an effective index of refraction ${\tilde{n}}_{2eff}=\frac{\left(m_{21}+m_{22}{\tilde{n}}_{3}\right)}{\left(m_{11}+m_{12}{\tilde{n}}_{3}\right)}$.

Therefore, Equations (5) are still valid, but they are the solution for an effective index of refraction that includes, for instance, the optical properties of the substrate, and this is the result of the superposition of several, if not infinite, single layers, each one of definite thickness and properties.

Looking for an explicit and numeric solution, the general case, however, has to be specialized. We make here further simplifications: the substrate is not absorbing, like a glass slide, and we consider only one intermediate layer, as well not absorbing. After the substitution with explicit values for the matrix elements:

(9) $$\left\{ \begin{array}{l} m_{11}=m_{22}=\cos(\beta )\\ m_{12}=-\frac{i}{n_{2}}\sin(\beta )\;;\;m_{21}=-i\;n_{2}\sin(\beta )\\ \beta =\frac{2\pi \;n_{2}\;d_{2}}{\lambda } \end{array}\right.$$

we can write explicitly the expression for ${\tilde{n}}_{2eff}$:

(10) $${\tilde{n}}_{2eff}=\frac{n_{3}\cos(\beta )-i\;n_{2}\sin(\beta )}{\cos(\beta )-i\;\frac{n_{3}}{n_{2}}\sin(\beta )}$$

The reflectance amplitude of Equation (7) in this particular case can be explicitly written:

(11) $$\tilde{r}=\frac{\left(n_{1}n_{2}-n_{2}n_{3})\cos(\beta )+i(n_{2}{}^{2}-n_{1}n_{3})\sin(\beta \right)}{\left(n_{1}n_{2}+n_{2}n_{3})\cos(\beta )-i(n_{2}{}^{2}+n_{1}n_{3})\sin(\beta \right)}$$

Now we are able to write the left sides of Equation (5) finding the real and the imaginary parts of ${\tilde{n}}_{2eff}$ as written in Equation (10):

(12) $$\left\{ \begin{array}{l} n_{2eff}=\frac{n_{2}{}^{2}n_{3}}{\cos{\left(\beta \right)}^{2}n_{2}{}^{2}+\sin{\left(\beta \right)}^{2}n_{3}{}^{2}}\\ k_{2eff}=\frac{1}{2}\frac{sin(2\beta )\;n_{2}(n_{3}{}^{2}-n_{2}{}^{2})}{cos{\left(\beta \right)}^{2}n_{2}{}^{2}+sin{\left(\beta \right)}^{2}n_{3}{}^{2}} \end{array}\right.$$

Note that the imaginary part is not trivially null also if we have supposed that the actual index of refraction of medium labelled 2 (in our example, a cell) has to be considered real.

In summary, reflectance data processed by the KK transformation produce the reflectivity phase. In turn, the latter, together with reflectance, allows us to compute the real and the imaginary parts of a pseudo refracting index, not at all coincident with the index of refraction of the layer representing our sample. It seems an intricate situation, but it can be actually solved with ease almost in an analytical way.

We now focus our attention on the first part of Equation (12). It represents an oscillating curve roughly centered around $n_{2}$, which in reality we do not know. It can be shown that maxima and minima of the expression in function of the wavelength λ, in which experimental data are commonly expressed, coincide with extremes of the trigonometric functions in Equation (12). Therefore, for those special values, we can easily evaluate the functions:

(13) $$\left\{ \begin{array}{ll} n_{2eff}=\frac{n_{2}{}^{2}}{n_{3}}, & \beta =\left(2p+1\right)\;\frac{\pi }{2}\\ n_{2eff}=n_{3}\;, & \beta =\;p\frac{\pi }{2} \end{array}\right.$$

with β defined within the Equation (9).

Note that, depending on the comparison between $n_{2}{}^{2}/n_{3}$ and $n_{3}$, the two extreme solutions can be swapped with reference to the respective value of β. But because we presume to know the value of $n_{3}$, and if everything has been computed correctly, we are able to assign one of the extremes at the right one of the two possibilities for β, the other giving us trivially the value of $n_{2}$.

Therefore, from Equation (13) we have obtained the first important result:

(14) $$n_{2}\left(\bar{\lambda }\right)=\sqrt{n_{3}\left(\bar{\lambda }\right)n_{2eff}\left(\bar{\lambda }\right)}$$

computed at a specific $\bar{\lambda }$.

Note that for no reason we have to assume that $n_{2}$ is wavelength independent, while actually we expect to see a more or less pronounced dispersion. Suppose to have computed the values of $n_{2}(\lambda )$ at two consequents maxima, or minima. We can write a couple of connected equations:

(15) $$\left\{ \begin{array}{l} \frac{2\pi n_{2}(\lambda_{1})\;d_{2}}{\lambda_{1}}=\left(2p+1\right)\frac{\pi }{2}\\ \frac{2\pi n_{2}(\lambda_{2})\;d_{2}}{\lambda_{2}}=\left(2p-1\right)\frac{\pi }{2} \end{array}\right.$$

We can then solve for the thickness *d* and for the order *p*:

(16) $$\left\{ \begin{array}{l} d_{2}=\frac{1}{2}\frac{\lambda_{1}\;\lambda_{2}}{\lambda_{2}\;n_{2}(\lambda_{1})-\lambda_{1}\;n_{2}(\lambda_{2})}\\ p=\frac{1}{2}\frac{\lambda_{2}\;n_{2}(\lambda_{1})+\lambda_{1}\;n_{2}(\lambda_{2})}{\lambda_{2}\;n_{2}(\lambda_{1})-\lambda_{1}\;n_{2}(\lambda_{2})} \end{array}\right.$$

The results shown in Equations (14) and (16) are of particular importance. Firstly, at specific wavelengths, trivially identified as the maximum, or the minimum, of the $n_{2eff}$ curve, we can deduce the thickness $d_{2}$ of our sample. Because the thickness is not wavelength dependent (if that is the case, we obtain a measure of the overall accuracy and of error propagation), and because we obtain that value for every point of our image, we have reached the remarkable result to have transformed a reflectance map into a true topographical image. That is just the aim of many interferential microscopies, but here it is used through a completely different and probably simpler technique. Moreover, at the same selected wavelengths we obtain the values of the real part of index of refraction $n_{2}$, again at every point of the explored surface, so we have, in addition, an RI map as well.

At this point it would be nice to trace $n_{2}\left(\lambda \right)$, the entire dispersion curve of the index of refraction. We have available maybe four or five wavelengths, depending on many maxima or minima are visible for the specific values of the various parameters. We can merely interpolate the available points, or we can revert to full numerical simulations that now, because the knowledge of the thickness $d_{2}$, are particularly simple.

However we can add a few other “special” points always on pure algebraic reasoning. The first of the Equation (12) has inflection points when the trigonometric parts have π/4 as their argument; in this case $n_{2eff}=\frac{2\;n_{2}{}^{2}\;n_{3}}{n_{2}{}^{2}+n_{3}{}^{2}}$. The specific point can be found approximately by curve inspection, more accurately by looking for the maxima in the associated differential curve, or observing that the curve for $k_{2eff}$ in Equation (12) has approximately the maxima and the minima in the right wavelengths positions. Therefore at those spectral positions we can add the values for the index of refraction:

(17) $$\left\{ \begin{array}{l} n_{2}\left(\bar{\lambda }\right)=\pm \;n_{3}\sqrt{\frac{n_{2eff}\left(\bar{\lambda }\right)}{2n_{3}-n_{2eff}\left(\bar{\lambda }\right)}}\\ \beta =\frac{2\pi n_{2}\left(\bar{\lambda }\right)d_{2}}{\bar{\lambda }}=\frac{\pi }{4} \end{array}\right.$$

Only the real and positive solution of the two expressions in Equation (17) has to be retained, of course.

### 2.2. Simplified Approach of Reflectance Manipulation

The presented discussion of the various terms is general, but has a potential not fully exploited. In fact, the advantage of adding one more quantity, the phase *θ*, has a complete reason in finding both the real and the imaginary parts of the local ${\tilde{n}}_{eff}$. This effort will be completed in a forthcoming paper, and practical consequences of that omission are detailed in the discussion section. For a biological sample, a first crude approximation is to disregard the optical absorption part, so that a further shortcut exists. We can directly expand Equation (11), bearing in mind that all the involved index of refractions are real:

(18) $$\left\{ \begin{array}{l} R=\frac{(n_{1}{}^{2}-n_{2}{}^{2})(n_{2}{}^{2}-n_{3}{}^{2})\cos^{2}(\beta )+{\left(n_{1}n_{3}-n_{2}{}^{2}\right)}^{2}}{(n_{1}{}^{2}-n_{2}{}^{2})(n_{2}{}^{2}-n_{3}{}^{2})\cos^{2}(\beta )+{\left(n_{1}n_{3}+n_{2}{}^{2}\right)}^{2}}\\ \beta =\frac{2\pi n_{2}d_{2}}{\lambda } \end{array}\right.$$

Also in this case it is clear that for *R* the extremes of the expression are for $\beta =\left(2p+1\right)\;\frac{\pi }{2},\;\beta =p\;\pi$, the first representing a minimum for the spectrum, the latter a maximum:

(19) $$\left\{ \begin{array}{ll} R_{\min}={\left(\frac{n_{1}-n_{2}{}^{2}}{n_{1}+n_{2}{}^{2}}\right)}^{2}, & \beta =(2p\;+1)\frac{\pi }{2}\\ R_{\max}={\left(\frac{n_{1}-n_{3}}{n_{1}+n_{3}}\right)}^{2}, & \beta =p\;\pi \end{array}\right.$$

**Figure 1:**
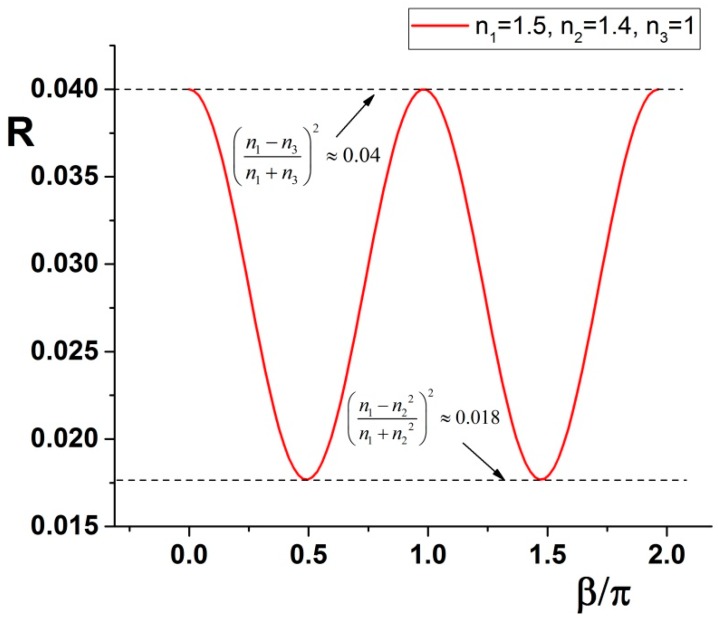
The reflectance *R* is plotted following Equation (18) having *n*_1_ = 1.5, *n*_2_ = 1.4, *n*_3_ = 1. The amplitude oscillates between the extremes described in Equation (19).

Putting some numbers, as an example for a situation described later and visible in Figure 1, we can trace the curve and see maxima and minima of the curve.

Note that the maximum of the *R* curve corresponds to a reflectivity for which the intermediate medium disappears completely. Conversely, the minimum of *R* it is not dependent on the last medium: this point allows evaluation of the local RI at that corresponding wavelength $n_{2}(\lambda )$. Combining then the information on the maxima and minima position it is possible to calculate the thickness of the medium 2. Explicitly, we have a solution for $n_{2}(\lambda )$:

(20) $$n_{2}=\sqrt{n_{1}}\frac{1+\sqrt{R_{\min}}}{\sqrt{1-R_{\min}}},$$

while for the thickness we can use the solution seen in Equation (16).

### 2.3. Fast KK Algorithm

Data obtained with the HSCM microscope are acquired as a stack of 16 bit unsigned data, with a separated record of the analogic range. The KK transformation has to be applied to every image point that, in this case, is actually an optical spectrum over, to say, *S* = 1000 wavelengths. Because the KK requires integration over all the spectral points, to be repeated for each one of the same points, the number of operations is of the order of *S*^2^. The integration has to be repeated for every *N*^2^ image points. For instance, if *N* = *512*, the total number of operations will be at least *KK_cycles_* = *S*^2^*N*^2^ machine cycles, that is *KK_cycles_* = 2.61 × 10^11^ cycles. Data are manipulated and imaged by a custom program written using C++, so the processor’s speed is reasonably well exploited. Generally speaking, it is required that, in any data elaboration, the overall delay will not impede a quite good interaction speed, if not a real time one.

The KK image transformation implies logarithms usage, for instance by using the *log*() included into the standard C++ libraries. Unfortunately that option is exceedingly slow. The claimed performances of a modern processor are of some GFlops/core, while a *log*() double precision single operation costs a lot of CPU cycles. Therefore the *log*() based mathematical operations, repeated *KK_cycles_* times, can require hours for a single image. While several smarter implementations are surely possible, another solution has been found.

The KK involves, in this particular case, the difference between two logarithms: $\log\left(R(\omega )/R(\omega_{0})\right)=\log\left(R(\omega )\right)-\log\left(R(\omega_{0})\right)$. Because data are memorized into two bytes integer, the actual spectrum values are quantized into 65,535 possible values. Therefore, before any calculation, a logarithm look-up table (LUT) is computed for any of the 16 bit allowable values, building something like an old logarithm book.

The logarithm difference is then quickly computed noting that the integer values, representing the specific spectrum points, are nothing more than a pointer to a value in the LUT, so that the difference is reduced to the floating point difference between two pointers at logarithmic values: $\log\left(R(\omega )\right)-\log\left(R(\omega_{0})\right)=(R(\omega )\to LUT\;)-(R(\omega_{0})\to LUT\;)$.

**Figure 2:**
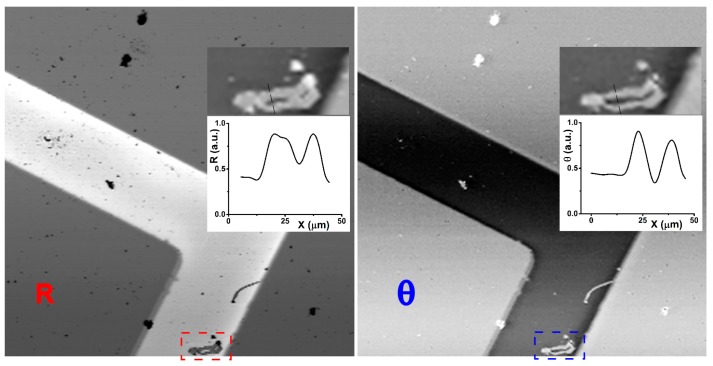
On the left, the hyperspectral reflectance confocal microscope (HSCM) image of a Au/Ti structure on a Si substrate is seen in a standard reflectance mode, shown for 600 nm wavelength. On the right, the correspondent phase image after the KK elaboration, selected for the same wavelength, shows rather clearer details, as it is clear looking to the defect marked (bottom) and visible magnified in the insert together with a detailed cross-section, for both images respectively. The reflectivity image part has been copied inverted to better compare with the phase image.

The required time ratio between the two approaches, as measured by a simple program on the same machine, is about a factor of 20. The KK is furtherly optimized splitting the calculations over all the available processor cores, because the KK transform can be parallelized on different image’s regions. At the end, an estimated time of 8 h for a single 512 × 512 pixel^2^ image (actually never tested till the end) reduces to a couple of minutes on an average PC. The effect of the KK elaboration on any image is straightforward. Also without any further elaboration, the phase image appears to give more natural visual information with a better 3D appearance, as it is well known as one of the advantages of a traditional phase-contrast microscope. An example image transformation is given in Figure 2 for a technological sample.

### 2.4. Sample Preparation and Geometry

**Figure 3:**
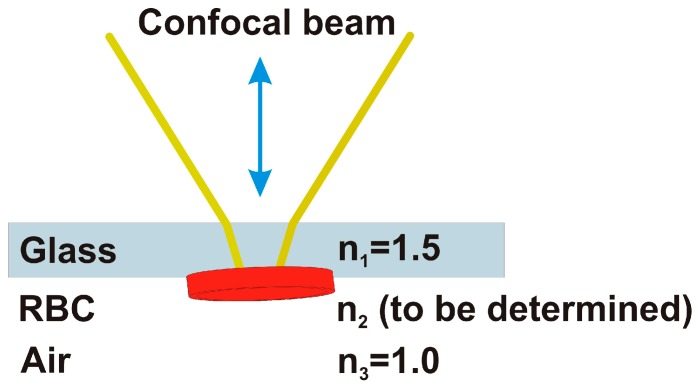
The incoming beam is propagating in air before entering the 1 mm thick glass slide. The focus is searched at the second glass-air interface where there are some of red blood cells (RBC). Radiation is partially reflected back forming the reflected image. All the wavelengths are focused on the specimen (white light), while the spectroscopic analysis is made by a couple of spectrometers after the focus on the output pinhole.

The RBC used here have been obtained spilling some blood drops, withdrawn from the author’s finger. The blood has been deposited on a microscope glass slide by rubbing directly the finger. The red cells are mounted as in Figure 3, with the beam penetrating firstly the glass slide, then impinging the cells, having the air then as the last medium.

In this way, the reflectance dielectric contrast is minimized, because it is between the glass $n_{1}=1.5$, and the biological specimen, for which usually $n_{2}\approx 1.3÷1.4$, like in an inverted microscope. In this case we can set $n_{3}=1$. The glass slide is kept lifted in order to avoid any contact of the red cells with the microscope sample holder.

## 3. Results

### RBC Data

**Figure 4:**
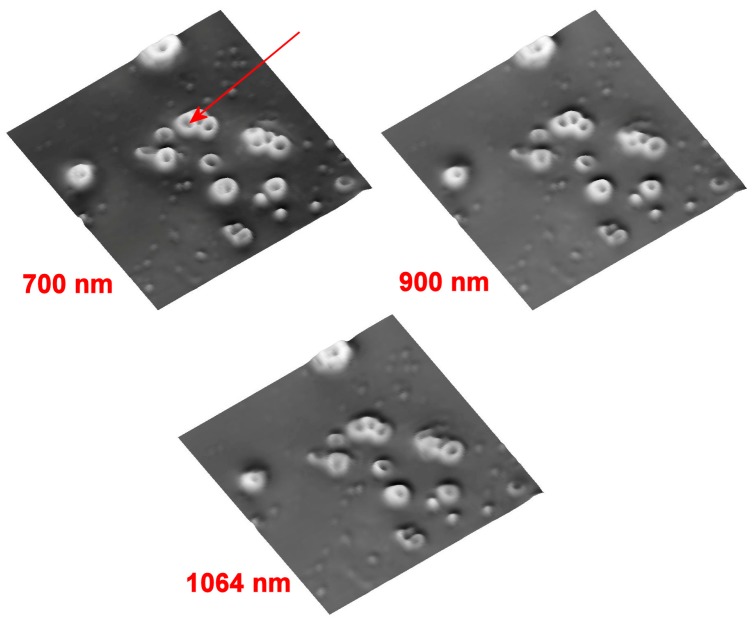
Reflectance HSCM image of a blood sample at 700 nm, 900 nm and 1064 nm, respectively. The overall intensities have been inverted to render the image more understandable. The red arrow shows the point where detailed plots are then given. The images, three of about 1000 acquired simultaneously, have a lateral size of 150 µm and are made by 512 × 512 pts^2^.

Preliminary images are taken to select the zone where one or more red cells appear clearly isolated with respect to the background. Finally, the selected field with a lateral size of 150 µm, 512 points × 512 points, is visible in Figure 4. It is possible to represent the data stack as a collection of images, one at each wavelength: on the left of Figure 4 the reflectance image for the 700 nm value is visible. The intensities have been inverted to obtain a more natural image, with the original reflectance data visible in the top-left insert. On the right, the same data are rendered as a 3D-image, showing the somewhat familiar shapes of erythrocytes, although we are dealing here with reflectance intensities, not true topography.

In this case, the whole stack of images is made by 1141 wavelengths between 500 nm and 2000 nm, while the intensity drops off heavily after 1600 nm. The wavelengths’ stack originates from the merge of two different detectors [1] with the IR part, the InGaAs array detector, made by a fixed set of 256 wavelengths, while the remaining part is coming from the CCD (Charge-Coupled Device) detector channels, reduced by a factor of 2 by hardware binning. The exit pinhole has a diameter of 100 µm. The full width at half maximum (FWHM) of the Z focusing curve is about 5.5 µm.

**Figure 5:**
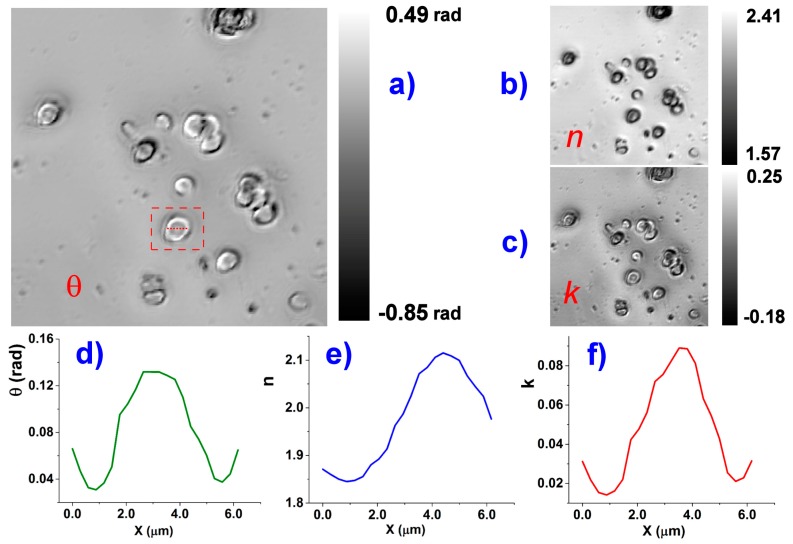
On the left is visible the RBC phase image (**a**); obtained by KK transformation of the HSCM image of Figure 4, at 700 nm. The image appears completely different from the correspondent reflectance image, while it is very similar to an ordinary phase-contrast optical microscopy images; On the right, the correspondent *n* and *k* images at 700 nm (**b**,**c**); The value bars, referred to the respective images, also include in the range image parts where the computed values have no sense, like the substrate or RBC boundaries for which the Fresnel expressions are not valid. An example of local values is given for an RBC marked of the left image: a cross-section is shown for the phase *θ* (**d**) and the components *n* and *k* (**e**,**f**) of the computed index of refraction. The values, however, have to be interpreted with the aid of the formalism in the text (e.g., see Equation (12)). The full wavelength dependence of *n* is accessible as a supplemental AVI file.

The KK transformation is applied to the whole image stack. A new triple stack is produced that includes the calculated wavelength dependent phase, the real and the imaginary parts of $n_{2eff}$, following Equations (4) and (12). The obtained images are visible in Figure 5.

**Figure 6:**
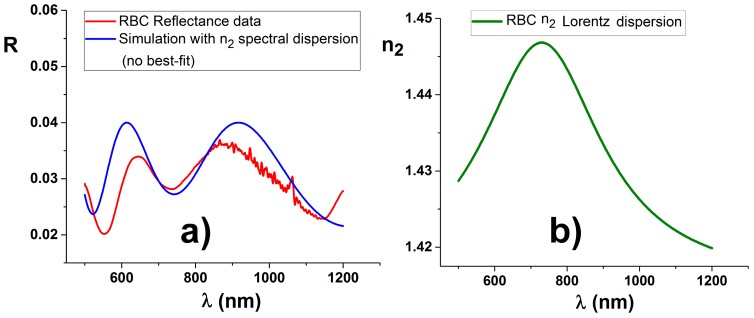
A reflectance curve, visible on the left (**a**), has been selected at the center of the RBC (red curve), marked in Figure 4. Solving procedures applied to the reflectance minima produce for *n*_2_ the values of 1.41, 1.45, and 1.43, respectively, with an averaged value of 1.43, while the evaluated thickness *d*_2_ is 750 nm ± 100 nm, both consistent with literature values. The overall shape is compatible with continuous *n*_2_ dispersion, represented by a Lorentzian shaped curve centered at 730 nm and with a width of 400 nm, visible on the right (**b**). No numerical best fit is used, but only a few values are chosen after some attempts. The normalizing ratio produce marked noise in the region around 1 µm (CCD section). The second detector data are not shown because simulation is unreliable in that region, due to accumulation of errors for the procedure of merging the two detector spectral regions.

As an example, we can use the reflectance data to show some parameters’ extraction. We can use the procedure described in the Equation (18) and the solutions defined in Equations (16), (19) and (20). In a future paper, extending the procedures to include full numerical approach, it will be possible to fit the whole curve and compute also the imaginary part of the local RI. The reflectance, as extracted from the position marked red in Figure 4, is plotted in Figure 6, together with an additional simulation.

The reflectance is normalized on the image itself with arbitrary selected points that clearly correspond to a free portion of the substrate. The ratio is then multiplied with the bare reflectivity of the interface, in this case $R_{0}=0.04$. While normalization is applied to every single point, the reflectance plots presented here are averaged over a circle of 10 pixels in diameter. The noisy points in reflectance of Figure 6, visible in 1000 nm region, correspond to the end of the CCD response, just as the first part of the InGaAs detector, so both tails are cut and the rest merged: the ideal combination between the two detectors is not optimally solved and will require a specific and improved software procedure.

Using the solutions as in Equation (20) for the three minima visible in Figure 6, it is possible to compute for *n*_2_ the values of 1.41, 1.45, and 1.43, respectively, with an averaged value of 1.43, while the evaluated thickness *d*_2_ is 750 nm ± 100 nm. Both values are consistent with literature values [30]. The overall shape is not simple like the one visible in Figure 1. However, it is compatible with dispersion for *n*_2_:

(21) $$n_{2}≅n_{0}+2\frac{A}{\pi }\frac{w}{4{\left(\lambda -\lambda_{c}\right)}^{2}+w^{2}}$$

with *n*_0_ = 1.415, *A* = 20, *w* = 400 nm, *λ_c_* = 730. Therefore a small dispersion in the red spectral region is found for better parametrize the RBC reflectance. However, obeying to the general aim we are following, no numerical fit is attempted to obtain optimized parameters.

## 4. Discussion

The aim of this paper is to show the adequateness of classical reflectance modelling for explaining at least a good part of the reflectance images obtained with a spectral confocal microscope, the HSCM. This explicit purpose has been partially or implicitly already demonstrated in the previously presented papers. In fact, in the first paper where the HSCM results have been shown [1], the layer models have been used for a numerical best fit of the measured reflectivity of golden structures of a technological test sample (a film made up of 60 nm Au/10 nm Ti over a Si wafer substrate), with good success.

On the other hand, none of the HSCM results for biological samples have been previously analyzed in this way [2,3,4]. Nevertheless, the correlation, statistically proven, between biological specimens and their evolution with reflectivity spectra show that reflectance features were found consistently over a number of cells within the same observation field, or among many different microscopy observation fields. However, because the possibility of using such models was temporarily pushed aside, the signal to noise ratio was often not good enough to make possible a new analysis of old data, but, at the same time, the overall shape of the observed reflectance showed the typical oscillations in the reflectance spectra explained here.

**Figure 7:**
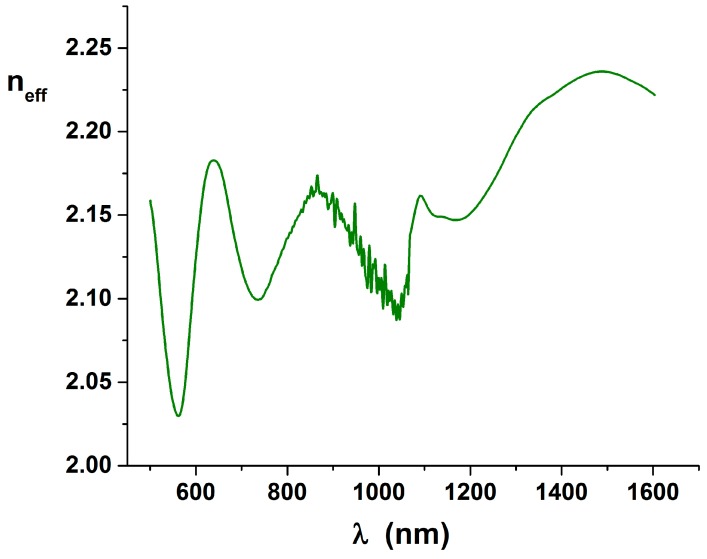
Plot of the *n_eff_* curve, derived from the set visible in Figure 5 and correspondent to the previously shown reflectance curve.

The presented material is only half of the work to be done. The approach used in this paper has been to give the best visibility of concepts, equations, and the very simple formalism in order to understand the structure of the observed data. Completing the model with, for instance, the explicit, numerical solution of the imaginary part of the unknown index of refraction (absorption) will require numerical solutions. Also the transformation of phase maps after a KK elaboration, producing RI maps or morphological thicknesses images, can be obtained only via numerical elaboration. In particular, it is possible to a posteriori justify why the method chosen to compute the RI for RBC has been the simplified one instead of the complete KK method. From the calculated set visible in Figure 5 ${\tilde{n}}_{eff}$ is derived and plotted in Figure 7.

It is clearly visible that the minima of the curve are at least shifted with respect to the values expected in Equation (13), because we expect that the minimum would be near $n_{3}\approx 1$. But we have seen that reflectance can be better explained by an additional small *n*_2_ dispersion that means that a correspondent imaginary *k*_2_ has to be expected. Because the KK equations are inherently bonding the real part with the imaginary part of the optical constants, the simplification made after Equation (8) are not valid, and the imaginary part has to be explicitly added in the definition of ${\tilde{n}}_{eff}$. The phase image can be only explained and used completing the work.

Some critical points also need to be addressed. RBC results have been used to show an application of the general methods, with no claim for new and better results for the very important RBC topic. While one typical RBC reflectance spectrum has been shown, spectra over other cells have some differences in amplitudes or shapes. To some extent, this can be attributed to the specific shape of RBC cells randomly oriented with respect to the beam. Also, because the sample preparation was not the best one possible, there is no warranty that there are no additional air gaps between the cell and the substrate. In fact, the simplest three layer model presented here is extremely well suited for technological samples, where locally flat and adherent films, also if of unknown thicknesses, are provided.

In the case of biological samples, instead, a suitable preparation is needed and advisable, for instance with the cells are as adherent as possible to their substrate. Also the liquid environment is a better choice because it eliminates the possibility of any gap between the first propagating medium and the specimens. However, using more layers, in the matrix modeling, the simulation will increase its complexity, but it could be better suited to follow a real session with biological samples.

A possible advantage of this machinery is that useful parameters can be gained from the observation of only one, single and isolated cell, in its natural state, without any external labeling, a goal that in itself worth the extra effort needed to be developed further.

## Supplementary Materials

Supplementary materials can be accessed at: http://www.mdpi.com/1420-3049/21/12/1727/s1.
